# Significantly improved cell affinity of polydimethylsiloxane enabled by a surface-modified strategy with chemical coupling

**DOI:** 10.1007/s10856-022-06690-3

**Published:** 2022-09-23

**Authors:** Li Rao, Yuqin Liu, Haihan Zhou

**Affiliations:** 1 Department of Geriatrics, Renmin Hospital of Wuhan University, Wuhan, 430060 China; 2 Key Laboratory of Chemical Biology and Molecular Engineering of Ministry of Education, Institute of Molecular Science, Shanxi University, Taiyuan, 030006 China

## Abstract

Polydimethylsiloxane (PDMS) is a commonly used insulation/packaging material for implantable neural electrodes. Nevertheless, the PDMS-initiated tissue response would lead to the deterioration of the electrode performances post-implantation, owing to its intrinsic hydrophobic and cell-repellent surface. The conventional physical coatings by hydrophilic hydrogels or bioactive molecules are unable to maintain during the long-term implantation due to their low stability by physical adhesion. In this work, we first anchor both hydrophilic polyethylene glycol (PEG) and bioactive molecule poly-L-lysine (PLL) on the PDMS surface by chemical coupling to change the PDMS surface from hydrophobic and cell-repellent to hydrophilic and cell-adhesive. XPS tests indicate the chemically coupled modification layers are stable on the PDMS surface after experiencing a harsh rinse process. Contact angle measurements show that the use of PEG 600 with the moderate molecular weight results in the highest hydrophilicity for the resulting PDMS-PEG-PLL. PC12 cell evaluation results exhibit that the PDMS-PEG-PLL with PEG 600 leads to significantly larger cell adhesion area, more neurite number, and longer neurite length than the PDMS. The PDMS-PEG-PLL with PEG 600 featuring stable modification layers, high hydrophilicity, and superior cell affinity has great potential in stabilizing the neural electrode-tissue interface for the long-term implantation.

**Figure:**
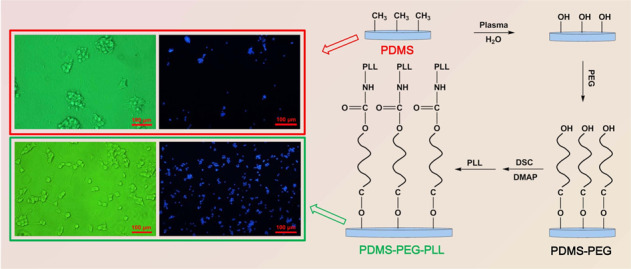
Graphical abstract

Graphical abstract

## Introduction

In recent years, conspicuous advancements have been achieved in the field of neuroprosthetic devices as neuroscience and microelectronics technologies develop. These devices are adopted to relieve symptoms or restore lost neurological functions by selectively stimulating the target neurons, such as cochlear implants that restore hearing to patients with damaged sensory hair cells, visual neuroprosthetic devices rebuilding vision to blind patients, and deep brain stimulators used to treat Parkinson’s disease and depressive disorder [1–4]. The neuroprosthetic devices are composed of electrical stimulators that transmit electrical pulse signal and implantable neural electrodes. The electrodes play a crucial role for neuroprosthetic devices because they directly communicate with the target neurons [5–7]. Nevertheless, the electrodes would initiate a tissue response, during which the glial scar generates and aggregates around the electrodes post-implantation. This will lead to the deterioration of electrode performances, because of the increased electrode impedance and decreased efficacy of electrical stimulation to the target neurons in the distance [8, 9]. Therefore, it is still a challenge to maintain the stable electrode performances in vivo over time. To this end, it is quite essential to establish an ideal electrode-tissue interface, for which the implanted electrodes elicit less tissue response and meanwhile neurons grow towards the electrodes. The electrode materials used are the key factor to determine the behaviors of the electrodes in vivo, including the tissue response initiated and affinity to neurons [10, 11].

Implantable neural electrodes consist of electronic conductors and insulation/packaging substrates. The former commonly employs the chemically inert materials like Pt, Au, and glassy carbon. The latter materials usually involve Si, polyimide, parylene-C, polydimethylsiloxane (PDMS), and so on [12–14]. Actually, the substrate part occupies the vast majority of area exposed to tissue relative to the conductor part in terms of the whole electrode. Thus the substrate materials adopted have a decisive influence on the behaviors of neural electrodes in vivo. PDMS is one of the most extensively used biomedical materials, because it features low cost, ease of fabrication, high flexibility, biological and chemical inertness, impermeability to water, permeability to oxygen, and nontoxic to cells, enabling a wide range of applications in cochlear implant, deep brain stimulator, vascular grafts, catheters, intraocular lenses, and contact lenses [15–18]. Unfortunately, the surface of PDMS is hydrophobic and cell-repellent, consequently going against establishing an ideal electrode-tissue interface.

Hitherto, different strategies have been explored to modify PDMS for constructing hydrophilic and cell-adhesive surfaces in various biomedical applications. Usually, two categories can be divided including physical coating and chemical coupling. For the former, physical coating with hydrogels was used to promote the hydrophilicity of PDMS for implantable neural electrodes, and physical coating with biomolecules like laminin and collagen was verified to improve the cell adhesion on the PDMS surface for microfluidic devices [19–22]. However, the physical coating is unable to maintain during the long-term implantation due to its low stability for physical adhesion. In contrast, chemical coupling is a promising method to construct a quite stable modification layer on PDMS surface due to the strong chemical bonding formed between them. In the previous studies, the bioactive molecules like extracellular matrix (ECM) proteins, polydopamine, and collagen were anchored on the PDMS surface by chemical coupling for improving the cell adhesion in microchips and cell sheet engineering [23–25]. Moreover, as mentioned above, it is anticipated to construct hydrophilic and cell-adhesive surfaces for PDMS to improving the electrode-tissue interface. Therefore, it is quite appealing to introduce hydrophilic molecules on the PDMS surface by chemical coupling in addition to bioactive molecules. To our knowledge, no study was performed to anchor both hydrophilic and bioactive molecules on the PDMS surface by chemical coupling for improving neural electrode-tissue interface.

In this study, we choose polyethylene glycol (PEG) and poly-L-lysine (PLL), serving as the hydrophilic and bioactive molecules, respectively. This is because PEG molecules show not only high hydrophilicity, but resistance to protein adsorption and platelet adhesion [26, 27]. PLL is an amino acid chain that is positively charged and commonly used as a coating to enhance cell adhesion for cell culture [28]. Herein, PEG and PLL were immobilized successively on the PDMS surface by chemical coupling. X-ray photoelectron spectroscopy (XPS) tests indicated that chemical coupling was a reliable method to maintain stable modification layers on the PDMS surface compared with physical coating method. The hydrophilicity of the resulting PDMS-PEG-PLL was optimized by using the PEG with different molecular weights. Contact angle measurements suggested PDMS-PEG-PLL with PEG 600 showed the smallest contact angle. The cell affinity was characterized by quantitatively evaluating the adhesion and differentiation of rat adrenal pheochromocytoma (PC12) cells on different materials. It should be noted that PC12 cell is a commonly used neuronal model which can extend neurites under the stimulation of nerve growth factor (NGF) [29, 30]. Cell evaluation results indicated that the cell affinity of PDMS was significantly improved after chemically coupling PEG and PLL, because the PDMS surface changes from hydrophobic and cell-repellent to hydrophilic and cell-adhesive.

## Materials and methods

### Materials

All chemicals and reagents were purchased from Sinopharm Chemical Reagent Co., Ltd (Shanghai, PR China), except for platinum silicone elastomer (A-103) and its cross-linking catalyst, which were purchased from Factor II, Inc. PC12 cells were purchased from the China Center for Type Culture Collection (Wuhan University). RPMI 1640 medium, horse serum, fetal bovine serum, penicillin, streptomycin, and l-glutamine were purchased from Thermo Fisher Scientific. Poly-L-lysine (PLL) (70,000–150,000 mol. wt.) and 4′,6-diamidino-2-phenylindole (DAPI) were purchased from Sigma. Nerve growth factor (NGF) was purchased from Wuhan Haite Biological Pharmaceutical Co., Ltd.

### The preparation of PDMS

Platinum silicone elastomer was mixed with its cross-linking catalyst at a ratio of 10:1 by weight and degassed under vacuum. Then PDMS thin films (~200 μm in thickness) were shaped with a stainless steel mold (12 cm in diameter) and cured at 80 °C in the oven for 2 h. After that, the PDMS films were cut into small pieces (~14 mm in diameter and ~30 mg in weight). Before use, the PDMS films were cleaned successively by acetone and ultrapure water under ultra-sonication, followed by drying at 40 °C, under vacuum for 12 h.

### The surface modification of PDMS

**Fig. 1:**
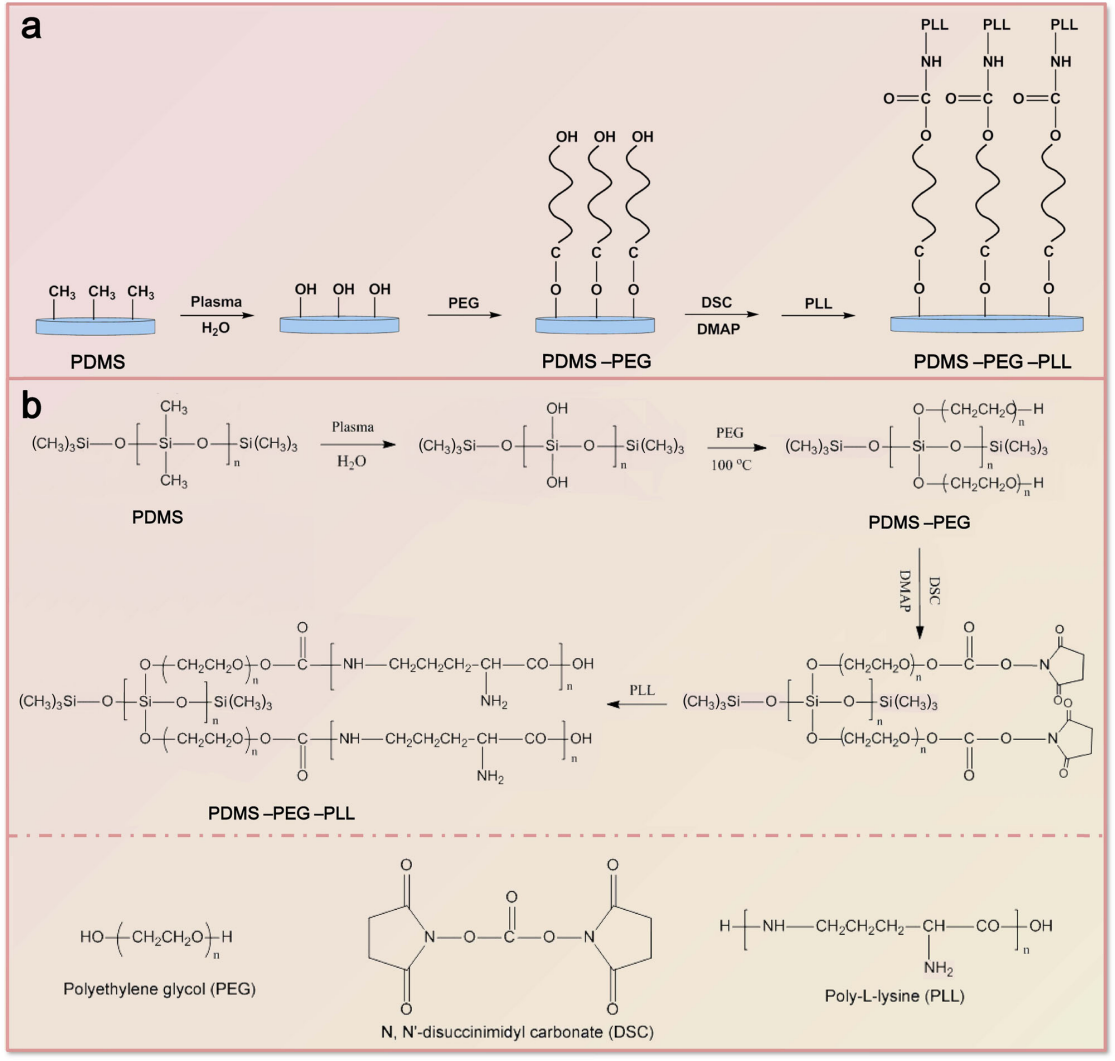
**a** Schematic diagram and **b** reaction process of surface-modified strategy to PDMS by chemically coupling PEG and PLL

As shown in Fig. 1a, the PDMS film was first treated by water plasma to transform the methyl on surface to hydroxyl group. A Corona Lab CTP-2000k plasma generator with 60 W was employed to perform the plasma treatment under a water vapor flow for 2 min. Subsequently, 0.1 M PEG solution was prepared by dissolving PEG in anhydrous N, N-dimethylformamide (DMF), and the PDMS films treated by plasma were immersed into the PEG solution, reacting in a drying oven at 100 °C for 24 h. Herein, the PEG with different molecular weights (*M*_w_ = 400, 600, 1000 and 2000) was used and labeled as PEG 400, PEG 600, PEG 1000, PEG 2000, respectively. After coupling with PEG, the resultant PDMS-PEG films were ultrasonically rinsed with DMF to remove unreacted PEG, and then immersed into a DMF solution containing 5 wt.% N, N′-disuccinimidyl carbonate (DSC) as the bridging agent, reacting in dark place for 24 h at room temperature with the 4-dimethylaminopyridine (DMAP) as the catalyst.

After being cleaned with deionized (DI) water, the DSC-treated PDMS-PEG samples were ultrasonically treated and placed at the bottom of 24-well tissue culture plate (TCP), with pristine PDMS as control. All the samples were dried for 0.5 h at 50 °C and then 600 μl 0.1 mg ml^−1^ PLL solution was added to the wells of TCP with samples, and without samples as the positive control, followed by incubating for 24 h at room temperature. Herein, 0.01 M phosphate-buffered saline (PBS, pH 7.4) was used to prepare PLL solution. During this period, the PLL was chemically coupled and physically coated on the DSC-treated PDMS-PEG and pristine PDMS, respectively. Correspondingly, the obtained samples were denoted as PDMS-PEG-PLL and PLL-coated PDMS, respectively. To compare the anchoring strength of PLL on the PDMS films, the PDMS-PEG-PLL and PLL-coated PDMS films were rinsed thoroughly with 0.01 M PBS before X-ray photoelectron spectroscopy (XPS) tests and cell culture. Specifically, all the samples were rinsed 8 times one day and last for a week.

### Material characterizations

Attenuated total reflection-Flourier transformed infrared (ATR-FTIR) tests were performed through a Thermo Nicolet Avatar 360 ATR-FTIR spectrometer. XPS spectra of samples were recorded by a Kratos XSAM 800 X-ray photoelectron spectrometer. A DSA100 Krüss goniometer was used to determine the water contact angle with a drop of ultrapure water, and six parallel samples were tested for each type of material (PDMS and PDMS-PEG-PLL). Data were exhibits by mean ± standard error of the mean (SEM).

### Cell adhesion evaluation

Cell adhesion and differentiation studies were performed to evaluate the cell affinity of these materials. For cell adhesion studies, PC12 cells were cultured in RPMI 1640 medium containing 10% of horse serum, 5% of fetal bovine serum, 2 mM l-glutamine, 100 U ml^−1^ of penicillin, and 100 μg ml^−1^ of streptomycin, under a humidified environment with 95% air and 5% CO_2_ at 37 °C. The replacement frequency of half the culture medium is two days. All samples placed in the TCPs with 24-well were sterilized by ethylene oxide gas before use. The seeding density of PC12 cells is 5 × 10^4^ cells for each well and they were incubated for 12 h. And then the plates were washed with PBS gently and the cells were stained with 0.5 μg ml^−1^ 4′,6-diamidino-2-phenylindole for 5 min. After staining, the cells were photographed using a Nikon Ti-S inverted fluorescence microscope equipped with a digital camera. Five random microscopic (×100) fields in each well were selected for each sample for analysis. The cell adhesion was assessed by calculating the relative area of PC12 cells on each substrate using Image-Pro Plus 6.0 (Media Cybernetics, Silver Spring). Each experiment was performed in quadruplicate culture wells. A paired Student’s *t*-test on SPSS software was used to compare different samples, and *p* < 0.05 represents statistical difference. Herein, PLL-coated PDMS samples and PDMS-PEG-PLL samples were rinsed thoroughly by 0.01 M PBS before the cell adhesion assays, to simulate the mechanical friction the electrode materials suffer from with the wet soft tissue in vivo.

### Cell differentiation evaluation

The PDMS samples used for cell differentiation were prepared in the same procedure with the cell adhesion experiment. Besides, TCPs were coated with the same amount of PLL, acting as a positive control. PC12 cells were subjected to starvation treatment after 12 h, and then induced to differentiate in starvation medium containing 50 ng/ml of NGF. After incubation for 7 days, at least eight images at ×200 for the PC12 cells in each well were acquired randomly by an inverted Nikon Ti–S phase-contrast microscope equipped with a digital camera, so that more than 50 differentiated cells were observed. The average number and length of neurites for each cell were used to quantify the differentiation level with the aid of Image-Pro Plus software. Triplicate culture wells were adopted for each experiment. Comparison between different substrates was made using paired Student’s *t*-test on SPSS software.

## Results

### Material characterizations

**Fig. 2:**
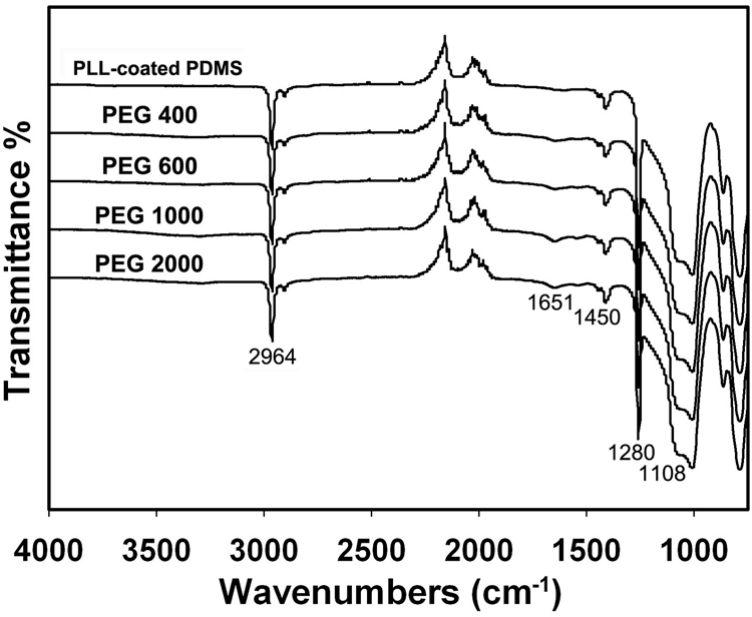
ATR-FTIR spectra of PLL-coated PDMS and PDMS-PEG-PLL with PEG at various molecular weights

The chemical structures of the PLL-coated PDMS and PDMS-PEG-PLL with PEG at various molecular weights were confirmed by ATR-FTIR. As shown in Fig. 2, all samples show strong absorption peaks located at 2964 cm^−1^, 1450 cm^−1^, and 1280 cm^−1^, attributed to the stretching vibration of N–H, absorption band of –NH–CO–, and bending vibration of N–H of amino groups in the PLL [31], respectively. The peak at 1108 cm^−1^ is assigned to the stretching vibration of Si–O bonds. Additionally, the peak at 1651 cm^−1^ is assigned to the –NH–COO– groups, confirming the reaction between the PEG chain with and amine groups of PLL.

**Fig. 3:**
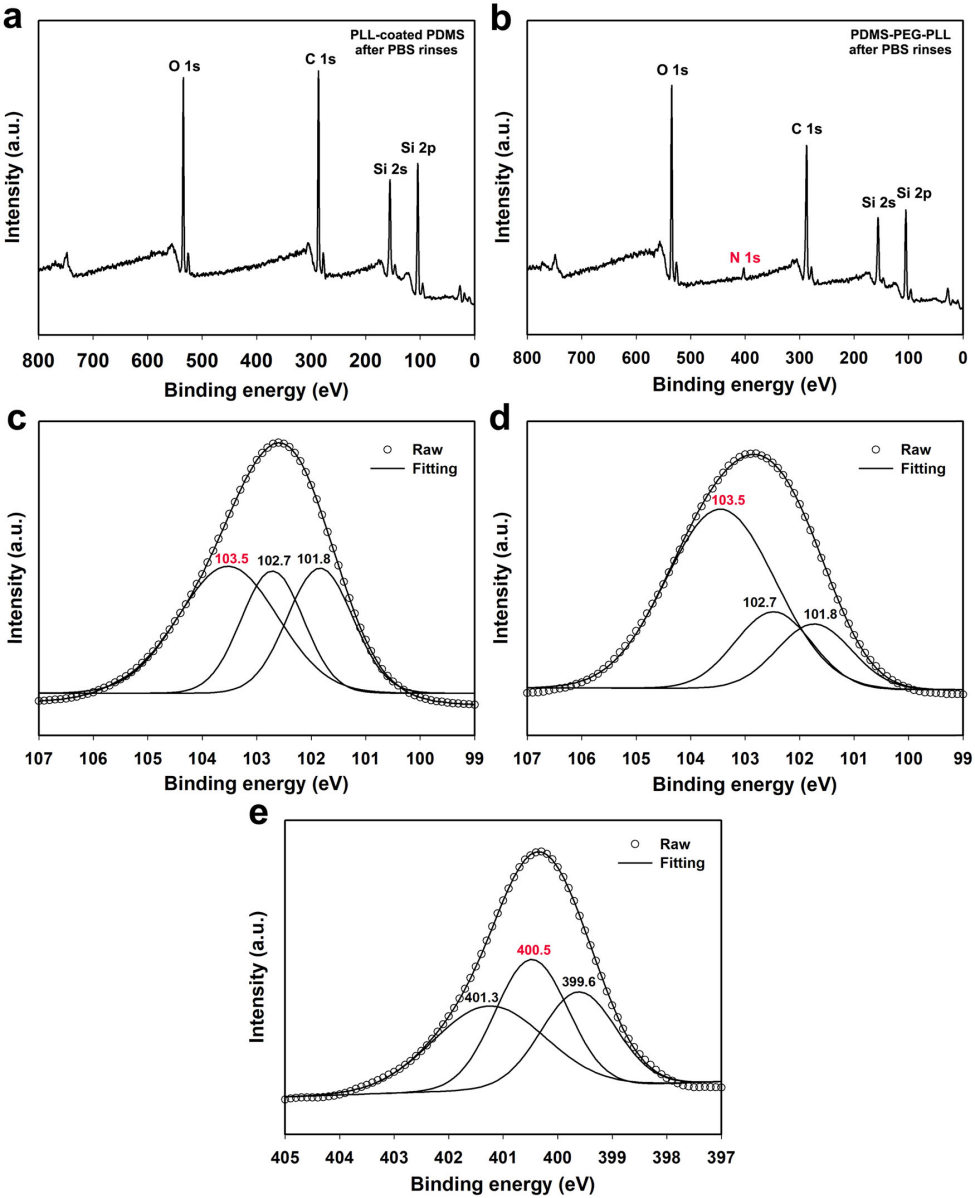
XPS survey spectra of **a** PLL-coated PDMS after PBS rinses and **b** PDMS-PEG-PLL with PEG 600 after PBS rinses. High-resolution Si 2*p* XPS spectra for **c** PDMS and **d** PDMS-PEG-PLL. **e** High-resolution N 1*s* XPS spectrum for PDMS-PEG-PLL

In order to confirm the superiority to immobilize the PLL on PDMS surface by chemical coupling, the PLL-coated PDMS and PDMS-PEG-PLL with PEG 600 were rinsed thoroughly by PBS solution before XPS tests. Figure 3a, b shows their XPS survey spectra after PBS rinses, respectively. Besides Si, C, and O elements, an additional XPS peak for N element can be observed for the latter, originated from the N in the PLL on PDMS, while no N element is detected for the former. Figure 3c, d exhibits the high-resolution Si 2*p* XPS spectra for PDMS and PDMS-PEG-PLL, respectively.

**Fig. 4:**
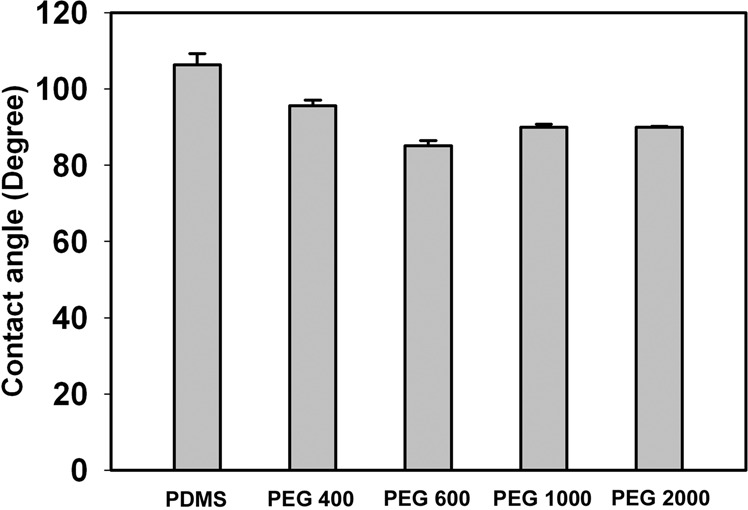
Water contact angles of PDMS and PDMS-PEG-PLL with PEG at various molecular weights. Data are exhibited by mean ± SEM (*n* = 6)

The water contact angles of PDMS-PEG-PLL with PEG at various molecular weights (PEG 400, PEG 600, PEG 1000, PEG 2000) are measured with the pristine PDMS for comparison. As shown in Fig. 4, the contact angle of the PDMS is 107°, while those decrease to 98°, 85°, 91°, and 93° for the PDMS-PEG-PLL with PEG 400, PEG 600, PEG 1000, and PEG 2000, respectively.

### Cell adhesion

**Fig. 5:**
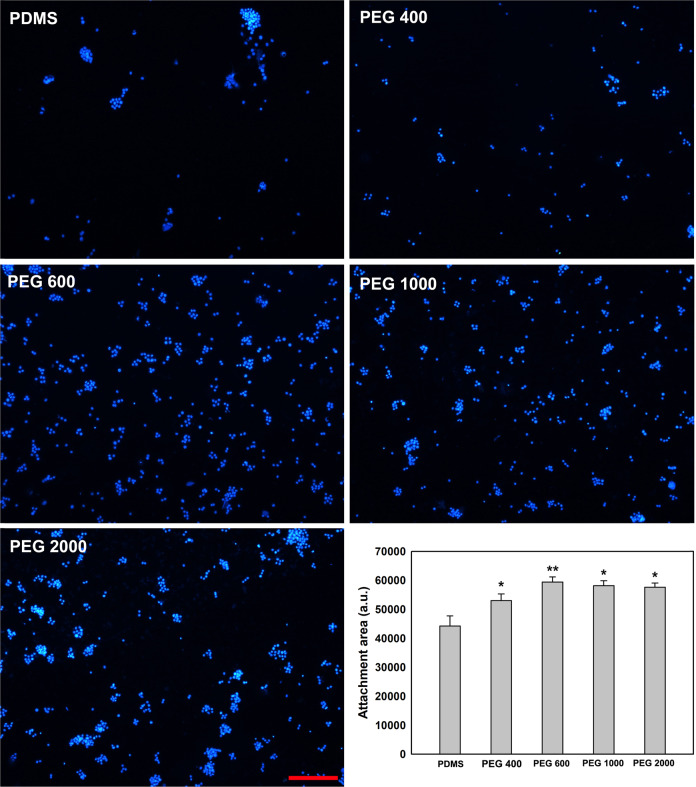
Representative cell adhesion images of PC12 cells stained by DAPI, cultured on PLL-coated PDMS and PDMS-PEG-PLL with PEG at various molecular weights after PBS rinses. The figure in the right bottom corner shows statistical PC12 cell adhesion area on PDMS and PDMS-PEG-PLL with PEG at various molecular weights. Data are shown by mean ± SEM (*n* = 12). Symbols * and ** represent the statistic difference (*p* < 0.05 and *p* < 0.01) in comparison to PDMS, respectively. Scale bar equals to 100 μm for all the images

Cell adhesion was used to evaluate the cell affinity of these materials. Because the repetitive PBS rinses lead to the complete removal of PLL coatings on the PDMS as verified by XPS tests in Fig. 3a, the PLL-coated PDMS after PBS rinses is abbreviated as PDMS in the following. As shown in Fig. 5, PC12 cells on PDMS are unevenly distributed and aggregate together as well as the cell density is extremely low. In contrast, after surface modification, the PC12 cells become evenly distributed and the cell density becomes higher for all PMDS-PEG-PLL with PEG 400, PEG 600, PEG 1000, and PEG 2000. Moreover, a quantitative statistical analysis of the photos of PC12 cell adhesion on these materials is performed, and the statistic results are shown in the bottom right corner of Fig. 5. It is obvious that the adhesion area of PC12 cells on all the PMDS-PEG-PLL samples is higher than that on PDMS, and presents statistic difference between all the PMDS-PEG-PLL and PDMS. Furthermore, for four types of PMDS-PEG-PLL, the order of cell adhesion area is PEG 600 > PEG 1000 > PEG 2000 > PEG 400.

### Cell differentiation

**Fig. 6:**
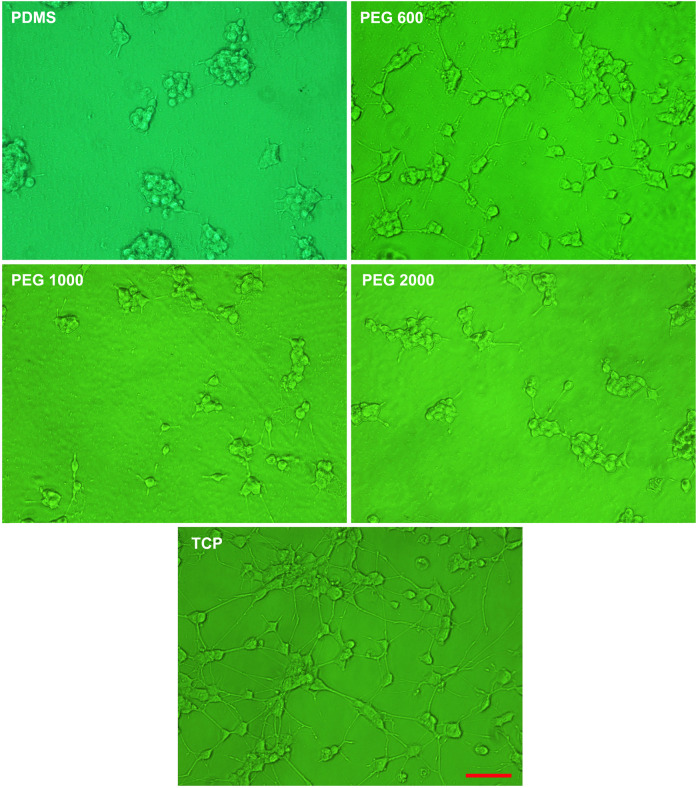
Representative cell differentiation images of PC12 cells on PDMS and PDMS-PEG-PLL with PEG at various molecular weights after PBS rinses. PC12 cells on TCPs act as the positive control. Scale bar equals to 100 μm for all the images

Since the adhesion of PC12 cells directly affects their subsequent proliferation and differentiation behaviors, the PDMS-PEG-PLL samples with PEG 600, PEG 1000, and PEG 2000 are further screened by investigating the differentiation of PC12 cells, because of their better cell adhesion behaviors than PEG 400. Herein, the PDMS-PEG-PLL samples with PEG 600, PEG 1000, and PEG 2000 act as the experimental groups, and PDMS and TCP serve as negative and positive control groups, respectively. As the control groups, it can be seen from Fig. 6 that the PC12 cells differentiated under the stimulation of NGF on PDMS are non-homogeneously dispersed and exhibit an undifferentiated round shape with few branching neurites, while PC12 cells on TCP are relatively homogeneously dispersed and more differentiated, forming a dense neurite network. Further observation manifests that the PC12 cells on PDMS-PEG-PLL with PEG 600, PEG 1000, and PEG 2000 exhibit improved differentiation behaviors relative to those on PDMS, because the former show the characteristics of dispersed distribution and formation of neurites.

**Fig. 7:**
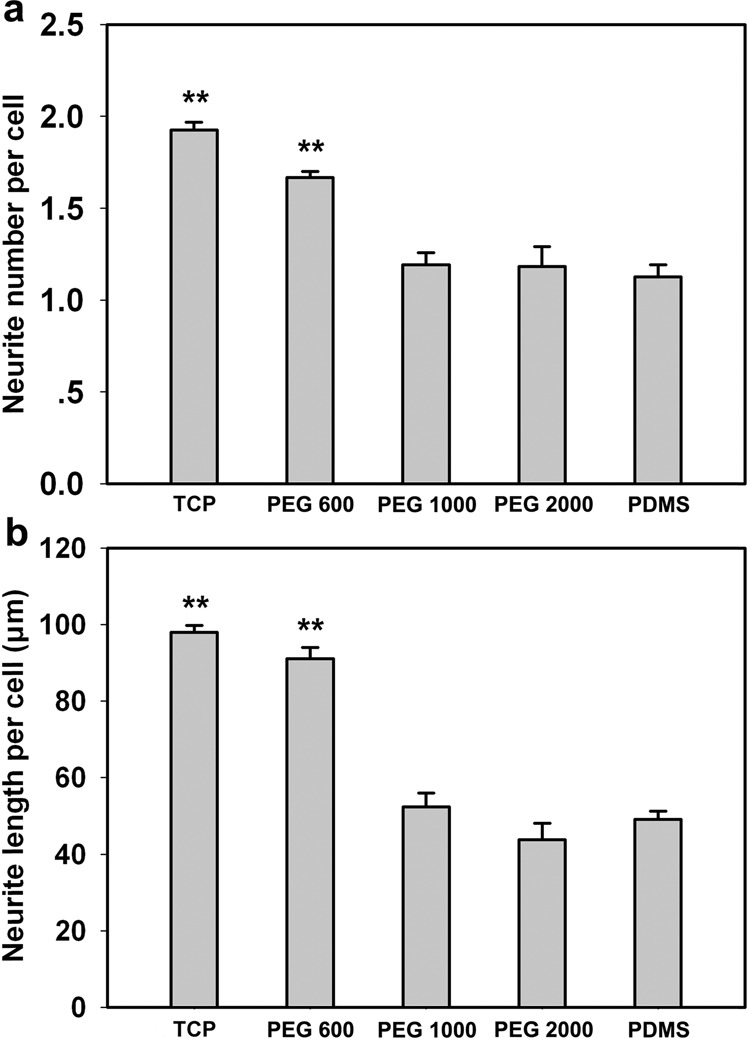
Statistical images of **a** neurite number for each PC12 cell and **b** neurite length for each PC12 cell on PDMS and PDMS-PEG-PLL with PEG at various molecular weights in the presence of NGF on day 7. PC12 cells cultured on TCPs act as the positive control. Data are presented by mean ± SEM (*n* = 12). Symbol ** represents the statistic difference (*p* < 0.01) in comparison to PDMS

In order to more accurately and objectively evaluate the differentiation of PC12 cells on different materials, a quantified method is used to evaluate the differentiation level by measuring the average neurite number and neurite length for each cell. Figure 7a exhibits that the average neurite number for each PC12 cell on PDMS-PEG-PLL is higher than that on PDMS, and PDMS-PEG-PLL with PEG 600 shows significant difference (*p* < 0.01) in comparison to PDMS. Moreover, the average neurite number for each PC12 cell on PEG 600 is comparable to that on TCP as the positive control. Figure 7b shows that the TCP and PEG 600 have close average neurite length per cell, which is observably longer than those on PEG 1000, PEG 2000, and PDMS. Also the neurite length per cell on PEG 600 is significantly higher (*p* < 0.01) compared to that on PDMS.

## Discussion

In order to confirm whether the PEG and PLL are successfully anchored on the surface of PDMS by chemical coupling, ATR-FTIR tests are employed to characterize PDMS-PEG-PLL with PEG at various molecular weights and the PLL-coated PDMS for comparison. Note that no chemical bonds are formed between PLL and PDMS for the PLL-coated PDMS. In contrast to PLL-coated PDMS spectrum, the enhancement in the peak intensity at 1108 cm^−1^ originating from the stretching vibration of Si–O bonds is because PEG is chemically coupled on PDMS (see Fig. 1b), resulting in the introduction of amounts of Si–O bonds [32, 33]. Furthermore, the additional peak at 1651 cm^−1^ due to –NH–COO– groups for the PDMS-PEG-PLL demonstrates that the end of PEG chain is chemically coupled with PLL (see Fig. 1b). The ATR-FTIR results testify the chemical coupling between PDMS and PEG as well as PEG and PLL.

The XPS tests suggest that the PLL coatings on PLL-coated PDMS were completely removed by the repetitive rinses. In contrast, PLL was still immobilized on the PDMS-PEG-PLL surface after being rinsed thoroughly. Refinement of Si 2p XPS peaks revealed that Si elements existed in three different chemical states. Correspondingly, the Si 2p peaks in both spectra are deconvoluted into three peaks centered at 101.8, 102.7, and 103.5 eV, which are attributed to (CH_3_)_3_Si–O–, –(CH_3_)_2_–SiO_2_– and (–CH_3_SiO_3_–, –SiO_4_–) bonds, respectively [34, 35]. It is obvious that the peak area of (–CH_3_SiO_3_–, –SiO_4_–) bonds at 103.5 eV in PDMS-PEG-PLL is substantially enlarged compared to that in PDMS. This could be ascribed to the fact that amounts of –CH_3_SiO_3_– and –SiO_4_– bonds are introduced when PEG is chemically coupled on PDMS. Figure 3e shows the high-resolution N 1*s* XPS spectrum for PDMS-PEG-PLL. It can be deconvoluted into three peaks situated at 399.6, 400.5, and 401.3 eV, corresponding to the N element in –NH–COC–, –NH–COO–, and NH_3_^+^, respectively. Thereinto, the generation of –NH–COO– bond at 400.5 eV indicates that the end of PEG chain is chemically coupled with PLL as shown by the structural formula in Fig. 1b. XPS results further suggest that the PEG and PLL are chemically coupled on the PDMS forming PDMS-PEG-PLL, agreeing well with the ATR-FTIR results. Here, we do not quantify the yields for every step, because the reactants are grafted on the surface of the PDMS substrate, and the product (PDMS-PEG-PLL) acts like a composite. In addition, the grafting mass of the PEG and PLL on the PDMS surface is very little relative to that of the PDMS films, and especially they can not be separated from the surface of the ‘composite’.

Many studies have shown that hydrophilic surfaces could reduce the inflammatory response elicited by implants [36–38]. To optimize the hydrophilicity of the resulting PDMS-PEG-PLL, the PEG at various molecular weights (PEG 400, PEG 600, PEG 1000, PEG 2000) was used, and the water contact angles are measured with the pristine PDMS for comparison. The results verify that the surface hydrophilicity of PDMS is obviously enhanced by the surface modification, which is due to the introduced oxygen-containing groups with high hydrophilicity in PEG. Among different PDMS-PEG-PLL samples, the PDMS-PEG-PLL with PEG 600 exhibits the smallest contact angle, indicative of its highest hydrophilicity. It’s generally thought that the hydrophilicity of PDMS-PEG-PLL depends on the amount of oxygen-containing groups (repeated –CH_2_–CH_2_O– units) provided by the anchored PEG on the PDMS. Nevertheless, the amount of the repeated –CH_2_–CH_2_O– units is related with two factors. One is the molecular weight of PEG itself, the other is the loading density of PEG on the PDMS. When the molecular weight of PEG is less than 600, the repeated -CH_2_-CH_2_O- units on the PDMS are mainly determined by the molecular weight of PEG itself because of little difference in the loading density of PEG on PDMS. Whereas, when the molecular weight of PEG is larger than 600, the steric-hindrance effect becomes obvious owing to their longer molecule chains. And then the loading density of PEG chains decreases as the molecular weight of PEG increases. Although the PEG 1000 and PEG 2000 have higher molecular weight, their lower loading density of PEG chains due to the steric-hindrance effect results in less repeated –CH_2_–CH_2_O– units coupled on PDMS than those provided by PEG 600. Therefore, among PDMS-PEG-PLL with PEG at various molecular weights, PDMS-PEG-PLL with PEG 600 shows the best hydrophilicity, which can be attributed to the moderate molecular weight of PEG and correspondingly moderate steric-hindrance effect.

PC12 cell can differentiate like nerve cells and form synapses with neighboring cells to build neural networks when stimulated by NGF. However, the precondition of PC12 cell differentiation is the cell adhesion on the materials [39]. Based on it, we first evaluate the cell adhesion behaviors of PC12 cells on different materials including PDMS and PDMS-PEG-PLL with PEG at various molecular weights.

As shown in Fig. 5, after modification, the PC12 cells become evenly distributed and the cell density becomes higher for all the PDMS-PEG-PLL. This can be attributed to the introduced PEG resulting in the hydrophilic surface for PDMS, which contributes to improving the cell affinity of the materials. Furthermore, for PDMS-PEG-PLL, PLL is still immobilized on the PDMS modified by chemical coupling due to the strong chemical bonds formed, even if they suffer from the repetitive PBS rinses. Thus the PLL on PMDS-PEG-PLL also enhances their cell affinity. This also indicates that chemical coupling is a stable method to maintain modification layers on the PDMS surface. In contrast, physical coating is unable to maintain during the long-term implantation due to its low stability by physical adsorption. Besides, quantitative analysis indicates that the adhesion area of PC12 cells on all the PMDS-PEG-PLL samples is higher than that on PDMS (see Fig. 5). For four types of PMDS-PEG-PLL, the order of cell adhesion area is PEG 600 > PEG 1000 > PEG 2000 > PEG 400. This observation is consistent with the results of the contact angles in Fig. 4, demonstrating that the cell affinity of the materials is closely related to their hydrophilicity.

Cell differentiation was also performed to evaluate the cell affinity of these materials. The results show that the PC12 cells on PDMS-PEG-PLL with PEG 600, PEG 1000 and PEG 2000 have improved differentiation behaviors relative to those on PDMS. Thereinto, PC12 cells on PEG 600 are well differentiated, having elongated neurites and more intricate neurite network similar to those on TCP, indicating that PDMS-PEG-PLL with PEG 600 has better cell affinity for PC12 cell growth and neurite outgrowth with respect to PDMS-PEG-PLL with PEG 1000 and PEG 2000, which is line with the cell adhesion results. Quantitative analysis of cell differentiation shows that the PC12 cells have more and longer neurites on PDMS-PEG-PLL with PEG 600 than on PDMS. This is likely attributed to the highest hydrophilicity shown by the PDMS-PEG-PLL with PEG 600. To sum up, PDMS-PEG-PLL samples with PEG 1000 and PEG 2000 show the similar cell differentiation behaviors with PDMS, while the cell differentiation behaviors on PDMS-PEG-PLL with PEG 600 are close to those on TCP. This suggests that PEG 600 is more suitable for the surface modification by chemical coupling to improve the cell affinity to PDMS.

## Conclusions

In summary, we put forward a surface-modified strategy with chemical coupling to anchor both hydrophilic PEG and bioactive molecule PLL on the PDMS surface, and the hydrophilicity and cell affinity of the resulting PDMS-PEG-PLL are optimized by comparing the PEG with different molecular weights used. Three key observations are made. First, the chemical coupling method we propose is verified to be a stable method to maintain modification layers on the PDMS surface in contrast to physical coating method. Second, the use of PEG 600 with the moderate molecular weight results in the highest hydrophilicity for the resulting PDMS-PEG-PLL. Third, the PDMS-PEG-PLL with PEG 600 shows significantly improved cell affinity based on PC12 cells relative to the PDMS, including larger cell adhesion area, more neurite number, and longer neurite length, because the PDMS surface changes from hydrophobic and cell-repellent to hydrophilic and cell-adhesive after the surface modification. The PDMS-PEG-PLL with PEG 600 featuring stable modification layers, high hydrophilicity, and superior cell affinity holds great promising in stabilizing the electrode-tissue interface for the long-term implantation of neural electrodes.
